# Bioactivation and Metabolism of Amino Acid MDMA Prodrugs in Zebrafish Embryos, Human Liver S9, Whole Blood, and Microdosed Human Urine

**DOI:** 10.1002/dta.70057

**Published:** 2026-03-15

**Authors:** Simon K. Wellenberg, Lea Wagmann, Matthias D. Kroesen, Philip Schippers, Matthias Grill, Jennifer Herrmann, Markus R. Meyer

**Affiliations:** ^1^ Department of Experimental and Clinical Toxicology and Pharmacology, Center for Molecular Signaling (PZMS), PharmaScienceHub (PSH) Saarland University Homburg Germany; ^2^ Department of Microbial Natural Products (MINS), Helmholtz Institute for Pharmaceutical Research Saarland (HIPS) Saarland University Saarbrücken Germany; ^3^ MiHKAL GmbH Allschwil Switzerland

**Keywords:** LC‐HRMS/MS, MDMA prodrug, metabolism, microdosing, pHLS9, zebrafish

## Abstract

3,4‐Methylenedioxymethamphetamine (MDMA) remains unapproved for therapeutic use despite the promising results of MDMA‐assisted psychotherapy. There is a need to better understand the safety, pharmacokinetics, and toxicology of possible MDMA‐based prodrugs. Like lisdexamfetamine, amino acid prodrugs of MDMA may enable more controlled systemic exposure, but their metabolic activation pathways and metabolites are not known yet. This study investigated the bioactivation and metabolism of the MDMA prodrugs, MDMA‐tryptophan (MDMA‐Trp), MDMA‐lysine (MDMA‐Lys), and MDMA‐glycine (MDMA‐Gly), in zebrafish embryos (ZE), pooled human liver S9 fraction (pHLS9), pooled fresh human whole blood (pFHWB), and human urine after microdosing (HMD). It elucidated mechanistic activation routes and identified screening targets relevant for drug testing and safety assessment. In ZE, MDMA‐Trp underwent hydroxylation and *N*‐dealkylation prior to amide cleavage, indicating a stepwise bioactivation pathway that differs from direct conversion observed for the other prodrugs. All three prodrugs were cleaved to MDMA in ZE, pHLS9, and HMD, with known MDMA metabolites additionally formed in ZE and pHLS9, whereas no metabolites were detected in pFHWB, suggesting that amide cleavage is not mediated in blood under the tested conditions. Unique urine screening targets were identified only for MDMA‐Trp, while biomarkers for MDMA‐Lys and MDMA‐Gly consisted of MDMA and known MDMA metabolites. This study demonstrated conversion of amino acid prodrugs to MDMA in pHLS9‐ and ZE‐based systems and in humans after microdosing, but not in blood. There is a need for further studies such as their pharmacokinetic profiles in humans.

## Graphical abstract

Bioactivation and metabolism of MDMA prodrugs: MDMA‐Trp, MDMA‐Lys, MDMA‐Gly showed unique urine screening targets only for MDMA‐Trp, while biomarkers for MDMA‐Lys and MDMA‐Gly consisted of MDMA and known MDMA metabolites. Conversion of amino acid prodrugs to MDMA in pHLS9, zebrafish embryos, and humans.

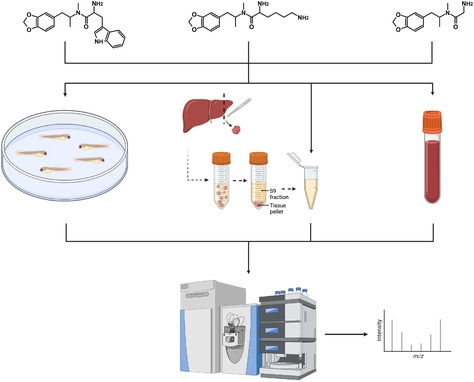

## 1 Introduction

3,4‐Methylenedioxymethamphetamine (MDMA) has long been considered a treatment option for post‐traumatic stress disorder and additional psychiatric conditions. However, approval for treatment was denied by the Food and Drug Agency (FDA) in 2024 due to concerns about the study design, drug safety, and possible abuse. There is still interest in addressing the FDA concerns to bring MDMA onto the market as studies have shown the benefits of MDMA‐assisted psychotherapy [1, 2]. An option is amino acid prodrugs, which are known to enhance bioavailability, safety profiles, and drug transport [3, 4]. A recent example is lisdexamfetamine, a prodrug of amphetamine linked to the amino acid lysine, which has lower abuse potential than its active metabolite amphetamine [5, 6]. Due to the described advantages of amino acid prodrugs, the question is whether the amino acid prodrug principle is also applicable for MDMA.

In a previous study, we developed a workflow consisting of a primary metabolism study in zebrafish embryos (ZE, *Danio rerio* ) followed by a confirmational metabolism study using human microdosing (HMD) [7]. ZE have long been utilized in metabolism studies due to their high concordance with human metabolism, which likely results from the presence of orthologue genes for approximately 70% of the human genome within the zebrafish genome [8]. Furthermore, experiments performed with ZE younger than 120 h postfertilization (hpf) are not classified as animal experiments under EU directive 2010/63/EU [9]. Therefore, this in vivo model can be employed without requiring additional ethical approval. Additional advantages lie within ease of handling and cost‐effectiveness. HMD experiments offer the advantage of delivering results for the species of highest interest, the human being, while being safe, cheap, quick, and easy to perform. Compounds are possible to administer in doses up to 100 μg while not exceeding the lowest pharmacologically active dose and a hundredth of the no observed adverse effect level [10]. In the current study, we aimed to complement this workflow by further metabolism models, pooled human liver S9 fraction (pHLS9), and pooled fresh human whole blood (pFHWB) for broader metabolite coverage [11, 12, 13]. This multimodel approach, ZE, HMD, pHLS9, and pFHWB, should allow us to investigate the amino acid MDMA prodrugs MDMA‐tryptophan (MDMA‐Trp), MDMA‐lysine (MDMA‐Lys), and MDMA‐glycine (MDMA‐Gly) across ZE, pHLS9, pFHWB, and HMD urine to define activation mechanisms and matrix‐specific screening biomarkers.

## 2 Materials and Methods

### 2.1 Chemicals and Reagents

MDMA‐Trp, MDMA‐Lys, and MDMA‐Gly (CAS Nr. 2763054‐43‐7, 2763054‐67‐5, 2763054‐39‐1, respectively) were provided by MiHKAL GmbH (Allschwil, Switzerland). Lisdexamfetamine dimesylate and trimipramine‐d_3_ were obtained from LGC GmbH (Luckenwalde, Germany). Sodium chloride (NaCl), potassium chloride (KCl), magnesium sulfate (MgSO_4_), calcium nitrate (Ca (NO_3_)_2_), and 4‐(2‐hydroxyethyl)‐1‐piperazineethanesulfonic acid (HEPES) were purchased from Carl Roth (Karlsruhe, Germany) and six‐well plates were from Greiner Bio‐One (Frickenhausen, Germany). ZE were obtained from in‐house breeding of adult zebrafish (AB wild‐type strain) at the Helmholtz Institute for Pharmaceutical Research Saarland. Eppendorf tubes and plastic urine cups were purchased from Sarstedt (Nümbrecht, Germany). Nicotinamide adenine dinucleotide phosphate (NADP^+^) was from Sigma‐Aldrich (Steinheim, Germany). 3′‐Phosphoadenosine‐5′‐phosphosulfate (PAPS), acetyl coenzyme A (AcCoA), dithiothreitol (DTT), isocitrate, isocitrate dehydrogenase, magnesium chloride (MgCl_2_), reduced glutathione (GSH), S‐(5′‐adenosyl)‐l‐methionine, superoxide dismutase were purchased from Merck KGaA (Darmstadt, Germany). UDP‐glucuronic acid 25 mM (UGT reaction mixture solution A), 125 μg/mL alamethicin (UGT reaction mixture B solution), and pHLS9 (20 mg microsomal protein/mL; 150 donors) were obtained from Discovery Life Sciences (Huntsville, LA, USA). pHLS9 was thawed at 37°C after delivery, aliquoted, snap‐frozen in liquid nitrogen and stored at −80°C until further use. HCX 3 mL/130 mg cartridges and C18 500 mg/3 mL cartridges were from Biotage (Uppsala, Sweden). Acetic acid, acetonitrile, ammonia, β‐glucuronidase/arylsulfatase (≥100,000 units/mL) from helix pomatia, dimethyl sulfoxide (DMSO), formic acid, hydrochloric acid, and methanol were obtained from VWR (Darmstadt, Germany).

### 2.2 Zebrafish Metabolism Experiments

#### 2.2.1 Zebrafish Maintenance

Husbandry of adult zebrafish was carried out in accordance with international guidelines and the German Animal Welfare Act (§11 Abs. 1 TierSchG). The experiments were performed with wild‐type ZE (AB strain) during the first 120 hpf, as experiments within these stages of development are not classified as animal experiments under EU Directive 2010/63/EU. ZE were kept and raised at 28°C in fresh Danieau's medium (17.4 mM NaCl, 0.21 mM KCl, 0.12 mM MgSO_4_, 0.18 mM Ca (NO_3_)_2_, 1.5 mM HEPES, and 1.2 μM methylene blue).

#### 2.2.2 ZE Maximum Tolerated Concentration Testing

Maximum tolerated concentration (MTC) testing was carried out based on the method described in a previous study [5]. ZE 4 dpf were transferred into six‐well plates, each plate containing 10 embryos and 3 mL of Danieau's medium containing different concentrations of each compound (0, 10, 25, 50, 75, or 100 μM, respectively) with 1% (V:V) DMSO. MTC testing was conducted in duplicate. The plates were incubated at 28°C for 24 h. Afterwards, survival rate, physical malformations, abnormal behavior, and heart rate of the embryos were evaluated using a LEICA M205 FA stereo microscope (Leica Mikrosysteme Vertrieb GmbH, Wetzlar, Germany).

#### 2.2.3 ZE Metabolism Study and Sample Preparation

The metabolism experiment was carried out as described in a previous study [5]. Embryos were exposed via waterborne exposure. The final concentrations were 100 μM for MDMA‐Trp and MDMA‐Gly and 75 μM for MDMA‐Lys. ZE 4 dpf were transferred into six‐well plates and 3 mL of Danieau's medium containing one compound and 1% (V:V) DMSO was added. Ten embryos were placed into each well and incubated at 28°C for 24 h. Afterwards, embryos from two wells were pooled. Experiments were conducted in duplicate for each compound. Furthermore, negative controls (Danieau's medium containing each compound and 1% (V:V) DMSO without ZE) and blank samples (ZE in Danieau's medium with 1% (V:V) DMSO without compound) were incubated at 28°C for 24 h.

After exposure, embryos were transferred into Eppendorf tubes and euthanized by placing the tubes on ice for 1 h. Embryos were washed twice with 1 mL of fresh and cold Danieau's medium, snap‐frozen, and lyophilized overnight. The lyophilizates were stored at −20°C for 24 h. ZE were extracted by adding 50 μL of methanol into the tubes, shaking for 2 min, and centrifugation at 18,407×*g* for 2 min. The supernatant was transferred into an autosampler vial and stored at −20°C for 1 day until analysis.

Danieau's medium used for the metabolism study was stored at −20°C for 1 day. For extraction, it was mixed with 50 μL of acetonitrile containing 0.1% (V:V) formic acid. This mixture was shaken for 2 min and cooled to −20°C for 30 min. Afterwards, it was centrifuged at 18,407×*g* for 2 min. The supernatant was transferred into an autosampler vial and stored at −20°C for 24 h until analysis.

### 2.3 Pooled Human Liver S9 Fraction Metabolism Experiment

The metabolism experiment with pHLS9 was performed according to established procedures with minor modifications [14]. First, 25 μg/mL alamethicin (UGT reaction mix B), pHLS9 (2 mg microsomal protein/mL), 0.1 mM AcCoA, 2.5 mM isocitrate, 8 U/mL isocitrate dehydrogenase, 100 U/mL superoxide dismutase, 0.6 mM NADP^+^, and 2.5 mM Mg^2+^ were preincubated at 37°C for 10 min. Afterwards, 2.5 mM UDP‐glucuronic acid (UGT reaction mix A), 40 μM PAPS, 1.2 mM SAM, 1 mM DTT, and 10 mM GSH were added. The reaction was initiated by adding the respective compound. In addition, negative controls without enzyme and blank samples without compound were incubated simultaneously. Incubations were performed in duplicate for each compound, and the concentration of organic solvent was kept below 1% [15]. Fifty‐microliter samples were taken at 60 and 360 min and the reaction was stopped by adding 30 μL of ice‐cold (−20°C) acetonitrile containing 2.5 μM trimipramine‐d_3_ as internal standard. The samples were then vortexed, stored at −20°C for 30 min and centrifuged at 18,407×*g* for 2 min. The supernatant was transferred into an autosampler vial and stored at −20°C until analysis.

### 2.4 Pooled Fresh Human Whole Blood Incubation

A total of 990 μL pooled (*n* = 4) fresh human whole blood (K_2_EDTA) were spiked with 10 μL of a 2.5‐mM solution of each compound respectively, resulting in a final concentration of 25 μM [16]. Incubations were performed in triplicate. In addition, positive control incubations with lisdexamfetamine (25 μM), negative controls without pFHWB and bank samples without substrate were performed. Fifty‐microliter aliquots were taken at 1, 15, 30, 60, 120, 180, and 240 min. Fifty microliters of ice‐cold (−20°C) acetonitrile containing the internal standard trimipramine‐d_3_ (2.5 μM) were added to stop the reaction and the mixtures were vortexed. Samples were then stored at −20°C for 30 min and afterwards centrifuged at 18,407×*g* for 2 min. The supernatant was transferred into an autosampler vial and stored at −20°C until analysis. The metabolic ratio was calculated as the full scan peak area of the active drug (amphetamine for lisdexamfetamine and MDMA for MDMA‐Trp, MDMA‐Lys, and MDMA‐Gly) divided by the full scan peak area of the corresponding prodrug.

### 2.5 HMD and Sample Preparation

The HMD metabolism experiment was done in accordance with a previous work with modifications [7]. All compounds were administered orally with at least 2 days between each compound. A dose corresponding to 100 μg of MDMA of each compound was dissolved in 1 mL of demineralized water and ingested. Spontaneous urine was collected before (*t* = 0) and after ingestion over 24 h and stored at −20°C until they were further processed. Samples were collected from one of the authors after 1.25 μg/kg bodyweight were applied for each compound. An ethics approval was not necessary for the HMD experiment as it is not required for self‐experiments in Germany.

For sample preparation, each urine sample was divided into two 3‐mL aliquots, one untreated and one undergoing conjugate cleavage. For conjugate cleavage, urine was adjusted to pH 5.2 with 1 M acetic acid and incubated at 50°C for 2 h with the addition of 50 μL of β‐glucuronidase/arylsulfatase solution. Both samples were then extracted via solid‐phase extraction, the untreated urine sample on a C18 cartridge and the processed sample on an HCX cartridge. The cartridges were preconditioned with 1 mL of methanol and 1 mL of demineralized water. Then, 3 mL of the samples were loaded onto the columns and afterwards, 1 mL of demineralized water followed by 1 mL of 0.01 M hydrochloric acid was added. In the following, vacuum was applied, and the cartridge was dried on the inside with pulp. After the addition of 2 mL of methanol, 1 mL of a freshly prepared methanol/ammonia (98:2, V:V) solution was added and eluted into an autosampler vial. The eluate was evaporated to dryness under nitrogen and reconstituted in 50 μL of methanol. Samples were then stored at −20°C until analysis.

### 2.6 LC‐HRMS/MS Setup

Analysis of all samples was done according to Gampfer et al. [17], using a Thermo Fisher Scientific (TF, Dreieich, Germany) Q Exactive Plus MS equipped with a heated electrospray ionization (HESI)‐II source and coupled to a Dionex UltiMate 3000 RS pump composed of a degasser, a quaternary pump, and an UltiMate autosampler. Mass calibration was done according to the manufacturer's recommendations prior to analysis using external mass calibration. An injection volume of 5 μL was used for all samples. Gradient elution was done on an Accucore Phenyl Hexyl Column (100 × 2.1 mm, 2.6 μm). Eluent A consisted of 2 mM aqueous ammonium formate solution with 0.1% formic acid (V:V) at pH 3. Eluent B consisted of 2 mM ammonium formate solution with acetonitrile/methanol (1:1, V:V), 1% demineralized water (V:V), and 0.1% formic acid (V:V). The flow rate started at 500 μL/min for 10 min and 800 μL/min from 10 to 13.5 min. The gradient was stepped from 0 to 1 min hold 99% A, 1–10 min to 1% A, 10–11.5 min hold 1% A, and 11.5–13.5 min hold 99% A. HESI‐II source settings were: heater temperature, 320°C; ion transfer capillary temperature, 320°C; spray voltage, 4.0 kV; ionization mode, positive and negative; sheath gas, 60 arbitrary units (AU); auxiliary gas, 10 AU; sweep gas, 0 AU; and S‐lens RF level, 50.0. Parent compounds and metabolites were putatively identified based on full scan data and data‐dependent MS^2^ (dd‐MS^2^) with an inclusion list, in which the masses of the parent compound and expected metabolites were included. MS^2^ spectra were compared to a library and if not included there, putatively identified by fragmentation patterns [18]. Full scan data acquisition was conducted as follows: resolution, 35,000; microscans, 1; automatic gain control (AGC) target, 1e^6^; maximum injection time (IT), 120 ms; and scan range, mass‐to‐charge‐ratio (*m*/*z*) 50–750. Settings for dd‐MS^2^ were as follows: option “pick others”, enabled; dynamic exclusion, disabled; resolution, 17,500; isolation window, 1.0 *m*/*z*; loop count, 5; AGC target, 2e^5^; maximum IT, 250 ms; high collision dissociation cell with stepped normalized collision energy, 17.5, 35.0, 52.5; exclude isotopes, on; spectrum data type, profile; and underfill ratio, 1%. Exact mass calculations and drawings of parent compounds and its metabolites was done with ChemSketch 2023.1.2 (ACD/Labs, Toronto, Canada). MS data files were handled with Thermo Xcalibur 4.6.67.17 QualBrowser.

## 3 Results and Discussion

In the following, results of metabolite ID in the different models will be discussed for each compound according to the order from the Methods section. Additionally, the HRMS/MS spectrum of each parent compound will be described exemplarily at the start of each subsection.

### 3.1 MDMA‐Tryptophan

**FIGURE 1:**
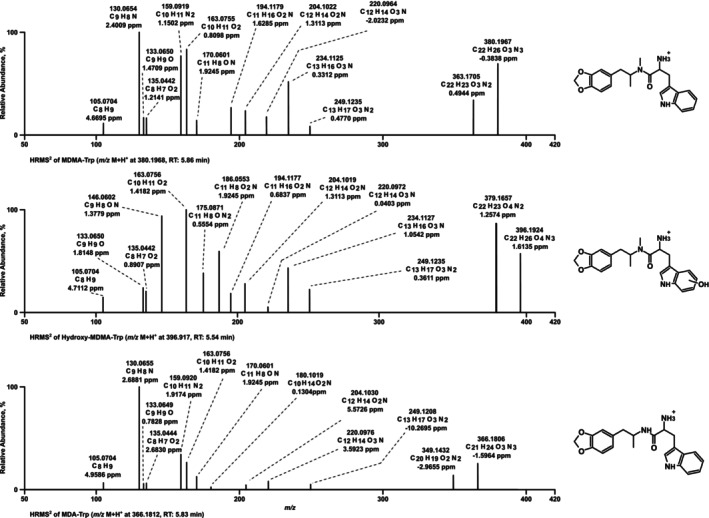
LC‐HRMS/MS spectra of MDMA‐Trp, hydroxy MDMA‐Trp, and MDA‐Trp measured in positive electrospray ionization mode; RT: retention time.

The HRMS/MS spectrum of MDMA‐Trp (Figure 1) showed the parent ion (PI) at *m*/*z* 380.1968 (C_22_H_26_O_3_N_3_+), which is followed by a fragment ion (FI) at *m*/*z* 363.1703 (C_22_H_23_O_3_N_2_+) resulting from the loss of ammonia (−17 u, NH_3_). The following FI at *m*/*z* 249.1233 (C_13_H_17_O_3_N_2_+) results from the MDMA residue after cleavage between the α‐ and the β‐carbon of the tryptophan side chain from the parent compound (−131 u, C_9_H_9_N), followed by the FI *m*/*z* 234.4457 (C_13_H_16_O_3_N+) after cleavage of an amine (−15 u, NH), the FI *m*/*z* 220.0968 (C_12_H_14_O_3_N+) resulting from cleavage of a methyl group (−14 u, CH_2_) and the FI *m*/*z* 204.1019 (C_12_H_14_O_2_N+) after loss of an oxygen atom (−16 u, O). The FI *m*/*z* 194.1175 (C_11_H_16_O_2_N+) corresponds to MDMA, followed by the described FIs of MDMA *m*/*z* 163.0753 (C_10_H_11_O_2_+), *m*/*z* 135.0440 (C_8_H_7_O_2_+), *m*/*z* 133.0647 (C_9_H_9_O+), and *m*/*z* 105.0698 (C_8_H_9_+). The FI at *m*/*z* 170.0600 (C_11_H_8_ON+) results from the tryptophan residue after amine loss and amide cleavage, the FI at *m*/*z* 159.0916 (C_10_H_11_N_2_+) corresponds to the tryptophan fragment after α‐cleavage, which is followed by an α‐cleavage at the aromatic system (*m*/*z* 130.0651; C_9_H_8_N+).

In the MTC testing, all embryos were found to be alive at a concentration of 100 μM and no physical malformations, abnormal behavior, or elevated heart rates were observed. A total of 100 μM could therefore be used in the ZE metabolism study.

**FIGURE 2:**
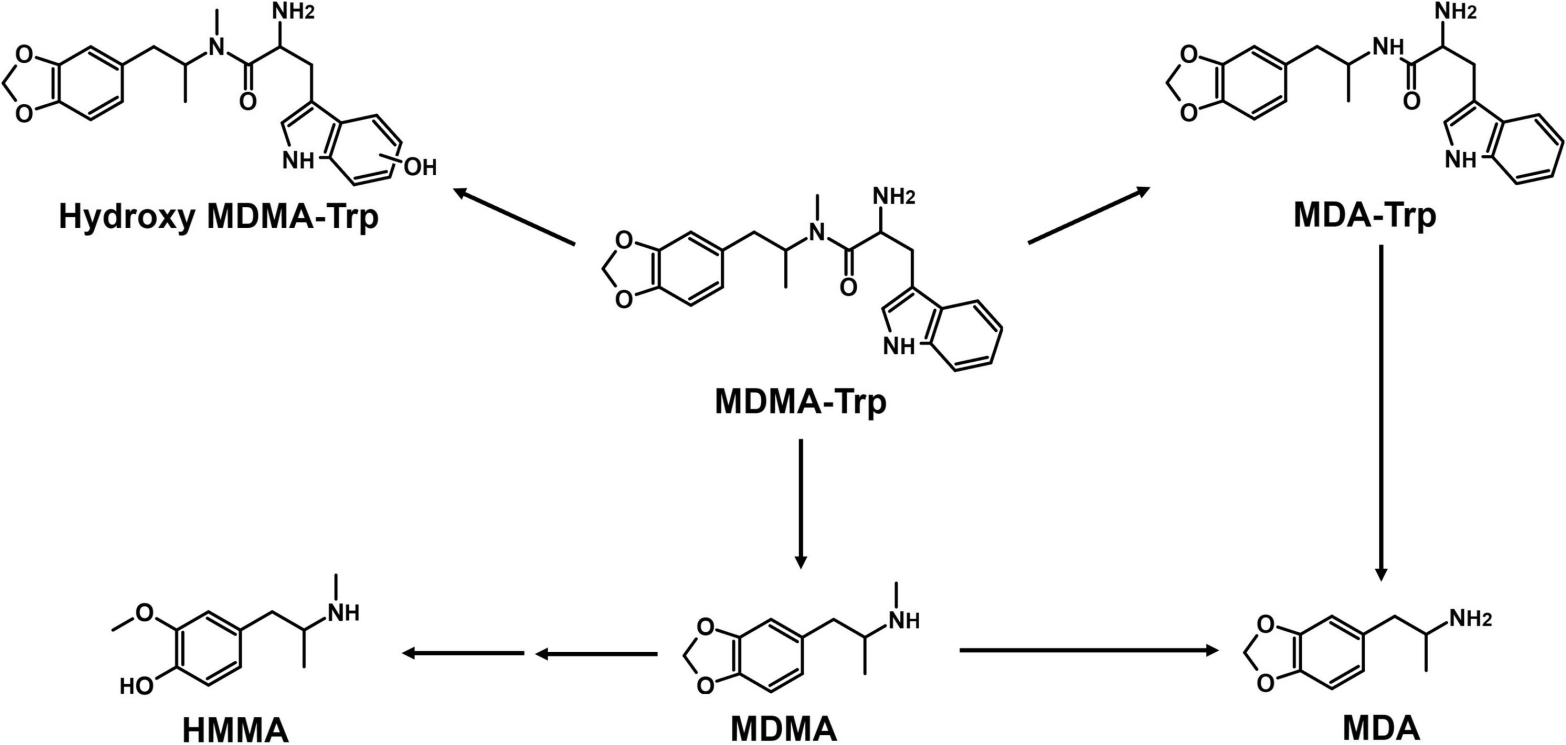
Proposed metabolic pathways of MDMA‐Trp; arrow: metabolized to.

As shown in Table 1 and Figure 2, MDMA‐Trp formed various metabolites. These included MDMA with its known follow‐up metabolites MDA and 4‐hydroxy‐3‐methoxy‐methamphetamine (HMMA). While MDMA was found throughout ZE, pHLS9, and HMD, its follow‐up metabolites could only be detected in ZE and pHLS9. Mass spectra of MDMA and its metabolites are not included in Figure 1 as they were already published previously [18]. Another common metabolite between ZE and pHLS9 was an aromatically hydroxylated metabolite at the tryptophan side chain (*m*/*z* 396.1917), indicated by the *m*/*z* shift of +16 for the tryptophan side chain fragment ions *m*/*z* 170.0600, 159.0916, and 130.0651 to *m*/*z* 186.0549, 175.0865, and 146.0600, respectively (see Figure 1). Furthermore, *N*‐dealkylation of MDMA‐Trp to MDA‐tryptophan (MDA‐Trp; *m*/*z* 366.1812) was detected in the ZE metabolism study only. However, apart from HMMA, no further phase II metabolites were found in the studies. In the pFHWB incubation (pFHWBI), neither amide cleavage nor any other metabolites were formed. Interestingly, the parent compound could not be detected in HMD samples. The proposed metabolic pathway is shown in Figure 2. None of the metabolites described above were detected in negative controls and blank samples.

**TABLE 1:** Marker of MDMA‐Trp, MDMA‐Lys, and MDMA‐Gly and their detection rate throughout the metabolism studies in ZE, pHLS9, pFP, pFHWB, and HMD; + = detected, − = not detected.

Compound	Metabolic marker	ZE	pHLS9	pFP	pFHWB	HMD
MDMA‐Trp	Parent	+	+	+	+	−
	HMMA	+	+	−	−	−
	Hydroxy MDMA‐Trp	+	+	−	−	−
	MDA	+	+	−	−	−
	MDA‐Trp	+	−	−	−	−
	MDMA	+	+	−	−	+
MDMA‐Lys	Parent	+	+	+	+	−
	HMMA	+	+	−	−	−
	HMMA sulfate	−	+	−	−	−
	MDA	+	+	−	−	−
	MDMA	+	+	−	−	+
MDMA‐Gly	Parent	+	+	+	+	−
	HMMA	+	+	−	−	−
	MDA	+	+	−	−	−
	MDMA	+	+	−	−	+

### 3.2 MDMA‐Lysine

**FIGURE 3:**
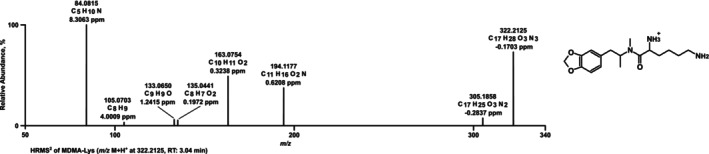
LC‐HRMS/MS spectrum of MDMA‐Lys measured in positive electrospray ionization mode; RT: retention time.

The HRMS/MS spectrum of MDMA‐Lys (Figure 3) showed a PI at *m*/*z* 322.2125 (C_17_H_28_O_3_N_3_+) followed by an FI at *m*/*z* 305.1859 (C_17_H_25_O_3_N_2_+) resulting from loss of ammonia. The next FI *m*/*z* 194.1175 (C_11_H_16_O_2_N+) corresponds to MDMA, followed by the described FIs of MDMA *m*/*z* 163.0753 (C_10_H_11_O_2_+), *m*/*z* 135.0440 (C_8_H_7_O_2_+), *m*/*z* 133.0647 (C_9_H_9_O+), and *m*/*z* 105.0698 (C_8_H_9_+). The last FI with *m*/*z* 84.0807 (C_5_H_10_N+) results from lysine after amine loss.

In MTC testing at a concentration of 75 μM, the survival rate was 100% and no abnormalities were observed. At 100 μM, 6 out of 20 ZE were found to be dead.

**FIGURE 4:**
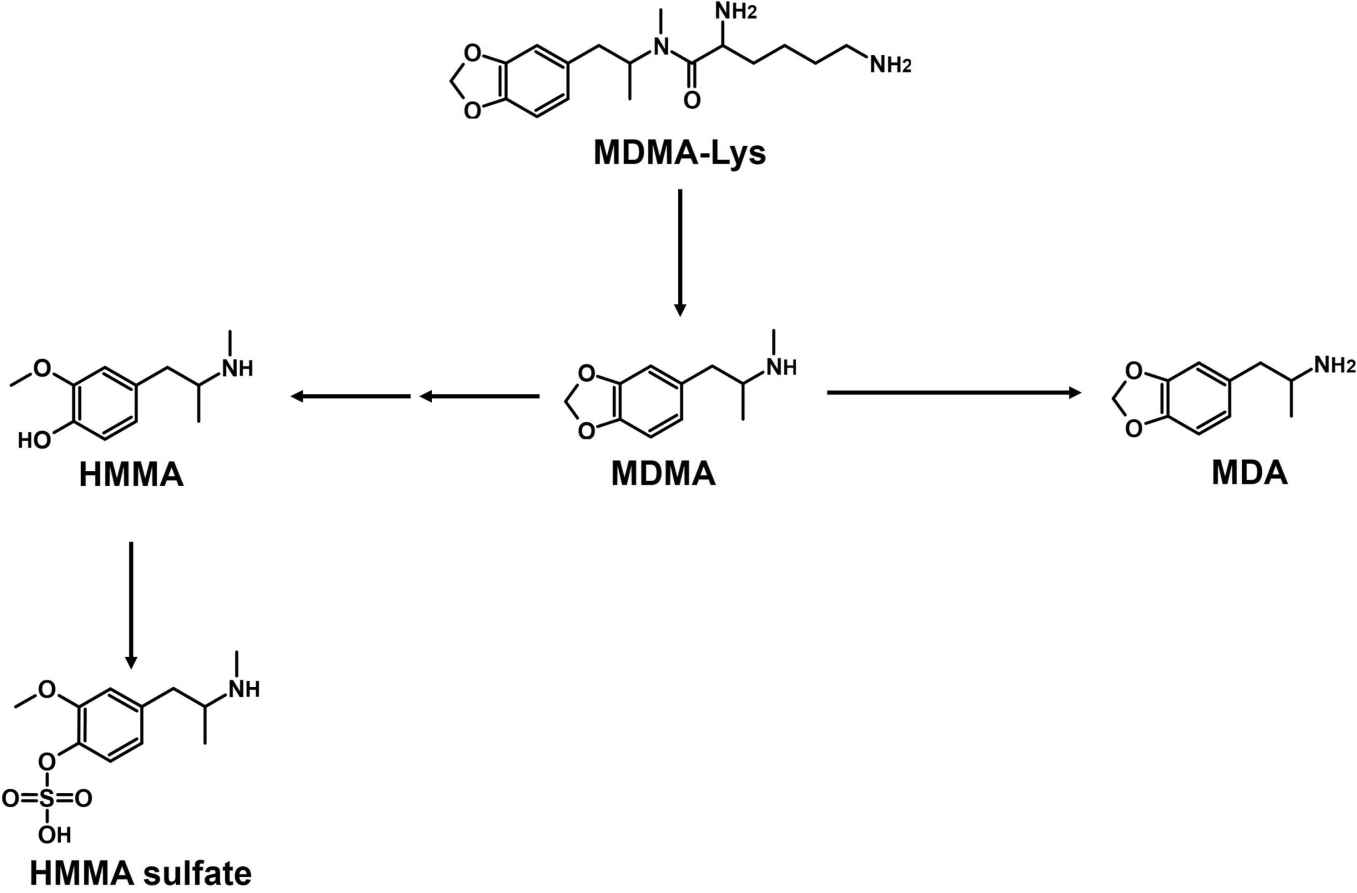
Proposed metabolic pathways of MDMA‐Lys; arrow: metabolized to.

MDMA‐Lys was cleaved in the ZE, pHLS9, and HMD studies but not in the pFHWBI study. MDMA was then further metabolized to MDA and HMMA in ZE and pHLS9. In contrast to MDMA‐Trp, HMMA sulfate was also found in pHLS9. Again, the HRMS/MS spectra of MDMA and its metabolites are not included in Figure 3 as they were described previously [18]. As also seen for MDMA‐Trp, the parent compound was not detected in HMD samples. The proposed metabolic pathway is depicted in Figure 4.

Blank samples and negative controls and the pure substance solution of MDMA‐Lys contained MDMA in small amounts. Due to higher abundance in experimental samples compared to negative controls and blank samples, MDMA was still included as a metabolite.

### 3.3 MDMA‐Glycine

**FIGURE 5:**
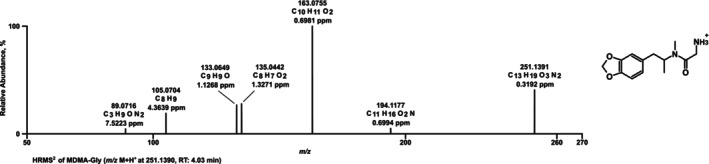
LC‐HRMS/MS spectrum of MDMA‐Gly measured in positive electrospray ionization mode; RT: retention time.

The LC‐HRMS/MS spectrum of MDMA‐Gly (Figure 5) showed a PI at *m*/*z* 251.1317 (C_13_H_19_O_3_N_2_+). The next FI *m*/*z* 194.1175 (C_11_H_16_O_2_N+) corresponds to MDMA, followed by the described FIs of MDMA *m*/*z* 163.0753 (C_10_H_11_O_2_+), *m*/*z* 135.0440 (C_8_H_7_O_2_+), *m*/*z* 133.0647 (C_9_H_9_O+), and *m*/*z* 105.0698 (C_8_H_9_+). The last FI at *m*/*z* 89.0709 (C_3_H_9_ON_2_+) corresponds to a 2‐amino‐*N*‐methylacetylamide ion. The MTC test led to the result that 100 μM MDMA‐Gly could be used for the ZE metabolism study as all ZE survived without behavioral changes or malformations.

**FIGURE 6:**
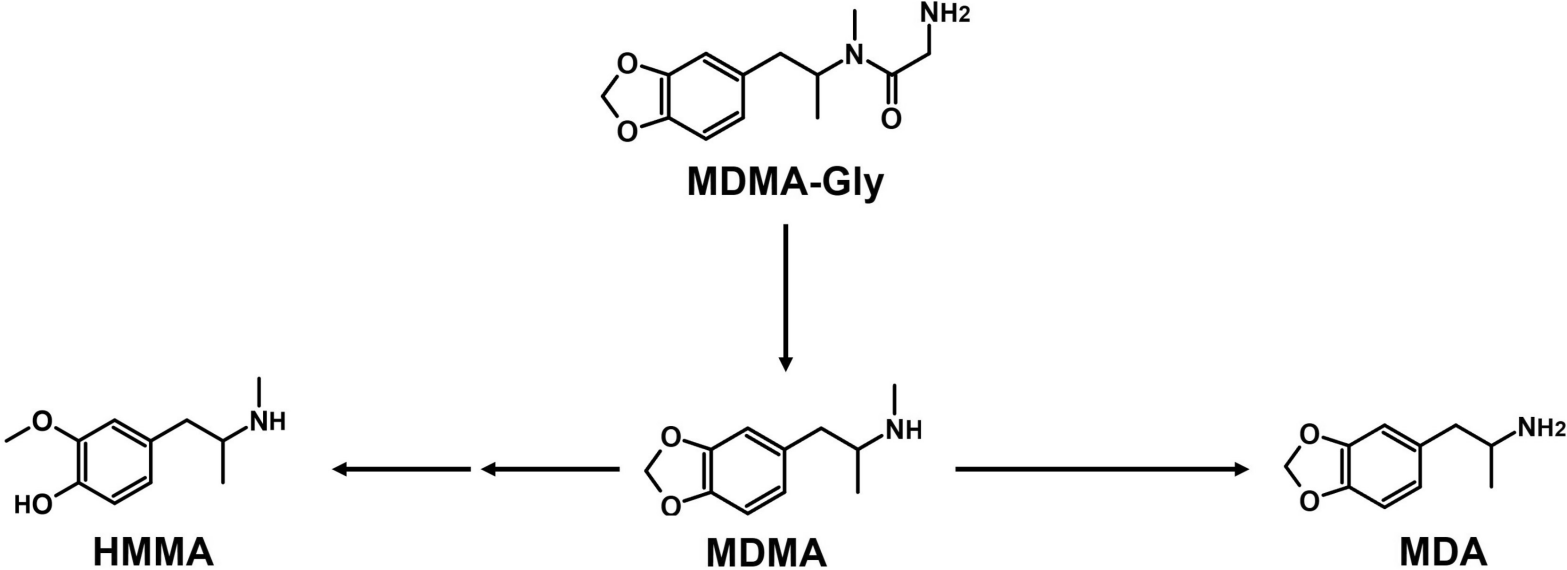
Proposed metabolic pathways of MDMA‐Gly; arrow: metabolized to.

MDMA‐Gly was once more successfully cleaved to MDMA in ZE, pHLS9, and HMD. In the ZE and pHLS9 study, MDA and HMMA were detected. Also here, the HRMS/MS spectra of MDMA and its metabolites are not included in Figure 5 as they were described previously [18]. However, no further metabolites of MDMA‐Gly itself or further phase II metabolites were detected in said metabolism models. The parent compound was once more not possible to detect in the HMD study. The proposed metabolic pathway is depicted in Figure 6. None of the metabolites described above were detected in negative controls and blank samples.

### 3.4 Metabolic Pathways, Metabolites, and Biomarker of Amino Acid MDMA Prodrugs

It was possible to identify a unique marker for MDMA‐Trp, namely, MDA‐Trp in ZE and hydroxy MDMA‐Trp in ZE and pHLS9, which are not described to be formed during intake of MDMA. Regarding the position of the hydroxylation of MDMA‐Trp, the analytical setup does not allow the determination of the exact position at the aromatic ring. However, hydroxylation in position 5, as typical in tryptophan metabolism, is most likely [19]. On the other hand, MDMA‐Lys and MDMA‐Gly did not produce any unique markers as it is also described for lisdexamfetamine [20]. Therefore, only a tryptophan side chain appears to be directly metabolized in contrast to MDMA‐Lys, MDA‐Gly, and lisdexamfetamine. All prodrugs were transformed into MDMA and metabolites of MDMA described in the literature even though conjugation with sulfate only occurred for MDMA‐Lys and glucuronidation could not be observed [21]. This may be due to the maximum incubation time of 24 h, which might explain the lack of conjugation following the expected delayed release of MDMA from the prodrug. If incubated or ingested for a longer period, further metabolites might have been formed.

Compared to our previous study [7], in which we exposed ZE to 100 μM MDMA via waterborne exposure, the intensity of MDMA‐derived metabolites was lower for the amino acid prodrugs in the present ZE study. This again implies the prodrug characteristic of the amino acid prodrugs. While the concentration of MDMA was 100 μM in both studies, lower intensities in the prodrug samples suggest that the release of MDMA might have been delayed. However, the ZE model does not depict temporal modulation with a singular sampling point. Therefore, the delayed release of MDMA is only hypothetical and not proven in this model. Furthermore, in the previous study, HMMA and MDA were possible to detect alongside MDMA in the HMD study. Combined with the finding from this study that only MDMA could be found, the expected delayed release of the active metabolite of the prodrug is most likely the reason.

Furthermore, results indicate that the amino acid prodrug principle seems to be applicable for MDMA as amide cleavage was possible in general. HMD data supported this finding as only the active metabolite, MDMA, could have been found in the urine samples. However, even though this finding suggests good prodrug capabilities, the low amount of compound applied in the HMD study needs to be critically considered as these low doses might lead to difficulties in detecting the parent compound and further (minor) metabolites. In addition, data from the HMD study are based on a single subject without pharmacokinetic profiling, which further limits generalization. Therefore, any conclusions drawn from the HMD experiment are of exploratory and hypothetical nature and need to be investigated in further controlled human studies. If higher doses were administered, more metabolites might have been found in human urine, such as MDA or HMMA or further phase II metabolites as described in the literature [22].

According to Schwaninger et al. [22], the onset of detection in urine after oral administration of 1 mg/kg or 1.6 mg/kg MDMA begins rather early for MDMA and its metabolites but the timepoint, at which the highest concentration was measured was often above 10 h after oral administration. However, the amount of MDMA administered in the study was eight hundred times the amount applied for the low dose of MDMA compared to the present HMD study (1000 μg/kg vs. 1.25 μg/kg). In addition, the expected delayed release of MDMA from the prodrugs will also delay the time of onset and maximum metabolite concentration.

**FIGURE 7:**
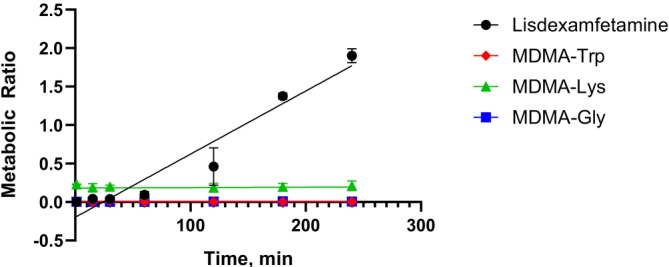
Results of the pooled whole human blood incubation for lisdexamfetamine (positive control), MDMA‐Trp, MDMA‐Lys, and MDMA‐Gly.

The lack of amide cleavage in plasma incubations might be due to the lack of amidases and peptidases. Even though amidases and peptidases are present in whole blood, amide cleavage was not detected in whole blood incubations, indicating that the cleavage will probably not take place in blood, at least during the 4‐h timespan. However, this should be further investigated, possibly including human blood samples following intake of an MDMA amino acid prodrug in the context of an HMD study. The incubation protocol was sufficient as there was an increase in the metabolic ratio for lisdexamfetamine (see Figure 7). The reason for the lack of amide cleavage for MDMA amino acid prodrugs compared to lisdexamfetamine might be caused by the additional methyl group at the amine, which might lead to steric hindrance.

## 4 Conclusions

Based on the results of this study, hydroxy MDMA‐Trp and MDA‐Trp might serve as markers for an MDMA‐Trp intake. MDMA‐Lys and MDMA‐Gly were not directly transformed into unique markers. However, MDMA and known MDMA metabolites were detected for MDMA‐Trp, MDMA‐Lys, and MDMA‐Gly. Interestingly, amide cleavage apparently does not take place in plasma or whole blood. Further studies should be conducted to investigate the pharmacokinetics of the prodrugs in human blood samples after HMD.

## Author Contributions

**Simon K. Wellenberg:** conceptualization, methodology, formal analysis, investigation, data curation, writing – original draft preparation, writing – review and editing, visualization. **Matthias D. Kroesen:** methodology, writing – review and editing. **Lea Wagmann:** conceptualization, methodology, writing – review and editing, supervision. **Philip Schippers:** methodology, writing – review and editing. **Jennifer Herrmann:** writing – review and editing. **Matthias Grill:** resources, writing – review and editing. **Markus R. Meyer:** conceptualization, methodology, formal analysis, investigation, writing – original draft preparation, writing – review and editing, visualization, supervision, project administration. All authors have read and agreed to the published version of the manuscript.

## Funding

The authors have nothing to report.

## Conflicts of Interest

The authors declare the following financial interest: Matthias Grill is CEO of MiHKAL GmbH. This does not alter our adherence to any policies on sharing data or material.

## Data Availability

The data that support the findings of this study are available from the corresponding author upon reasonable request.
